# Response of the tomato leaf miner *Phthorimaea absoluta* to wild and domesticated tomato genotypes

**DOI:** 10.1002/ps.8534

**Published:** 2024-11-12

**Authors:** Ayomide Joseph Zannou, Jörg Romeis, Jana Collatz

**Affiliations:** ^1^ Agroscope, Research Division Agroecology and Environment Zurich Switzerland

**Keywords:** *Tuta absoluta*, host plant resistance, trichomes, antixenosis, antibiosis

## Abstract

**BACKGROUND:**

*Phthorimaea absoluta*, a highly destructive invasive pest, poses a significant threat to tomato production globally. Exploring alternative control methods, such as host plant resistance can contribute to diminish reliance on insecticides and promote sustainable integrated pest management (IPM) practices. Thus, the identification of new *P. absoluta*‐resistant tomato cultivars and potential wild sources for breeding programmes remains imperative. We evaluated the effect of 19 tomato genotypes, comprising 16 domesticated varieties and three wild tomato species, on oviposition output of female *P. absoluta*, as well as on larval performance under no‐choice conditions using detached leaves. We also characterized and quantified glandular and nonglandular trichomes, exploring their potential correlation with the response of *P. absoluta* to the tomato plants.

**RESULTS:**

Generally, fewer eggs were oviposited on domesticated plants, whereas the wild tomatoes *Solanum arcanum* and *S. neorickii* and the domesticated tomato Corona F1 impaired larval development. The pest larvae consumed a limited area of leaflets from *S. arcanum* and *S. neorickii* compared to other genotypes, leading to the lowest weights in both male and female pupae. All tomato plants exhibited a prevalence of nonglandular over glandular trichomes, except for *S. arcanum*, which exhibited a higher abundance of glandular trichomes. Although higher trichome density correlated with longer larval settlement on the leaflets, it did not influence female oviposition.

**CONCLUSION:**

Our findings demonstrate that the wild tomatoes *S. arcanum* and *S. neorickii* could be considered as potential sources for breeding programmes, and the domesticated Corona F1 could offer IPM options against *P. absoluta*. © 2024 The Author(s). *Pest Management Science* published by John Wiley & Sons Ltd on behalf of Society of Chemical Industry.

## Graphical abstract

We evaluated oviposition and larval performance of *P. absoluta* on 19 tomato genotypes as an element of integrated pest management. Two wild tomatoes and a domesticated one showed resistance against the pest.

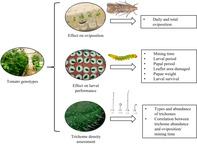

## 1 INTRODUCTION

The coevolution of plants and herbivores has resulted in the development of strong defence systems in plants to hamper herbivore attacks or to develop tolerance strategies.^1^ Host plants exhibit direct and indirect defence mechanisms in response to insect herbivores. Direct defence is expressed by physical barriers (e.g. trichomes, spinescence, waxy cuticles, sclerophylly) and secondary metabolism for the production of plant secondary compounds (e.g. toxins), which can be produced constitutively or induced by insect damage.^2^, ^3^ Indirect defence mainly occurs after attacks by insects (oviposition or herbivory) with the production of volatiles that attract and/or arrest natural enemies of the herbivores.^4^ These direct and indirect defence mechanisms facilitate crop resistance to herbivores by deterring pest landing behaviour, preventing attachment and feeding, reducing plant suitability or alerting and activating neighbouring plant defence, and attracting natural enemies of the target pests.^5^, ^6^ The additive effects of direct and indirect defence mechanisms have a strong impact on plant vigour and provide durable resistance to a broad spectrum of insect herbivores.^7^, ^8^

Host plant resistance has been used for decades as a single tactic or incorporated in an integrated approach to mitigate the pressure of herbivorous arthropods.^9^ Host plants can exhibit resistance to herbivory through antixenosis, antibiosis and tolerance.^10^ Antixenosis affects pest behaviour by reducing female oviposition and larval settlement, and antibiosis affects the life history of the pest by inhibiting larval and pupal development and limiting the emergence of offspring. By contrast, tolerance reduces the cost of herbivory‐induced wounds by being able to withstand or recover from damage caused by herbivorous arthropods.^10^, ^11^ However, the intensity of such defences is strongly related to the genetic background of host plants (i.e. domesticated plants or wild relatives).^12^, ^13^ Domesticated plants were often bred with the aim to provide enhanced nutritional content to better meet human needs, and tend to have simpler morphological traits and altered plant defence mechanisms, whereas wild plants have diverse morphological and chemical traits with strong defence mechanisms.^12^, ^13^, ^14^ To ensure optimal production in agriculture with lower reliance on insecticides, breeding programmes must consider both nutritional and defence aspects.^15^, ^16^ Tomato plants (*Solanum lycopersicum* L.) possess a combination of morphological and chemical traits that promote their defence against insect herbivores.^17^ This includes an array of primary and secondary metabolites and a variety of glandular and nonglandular trichomes.^18^, ^19^ Additionally, tomato plants respond to herbivory by releasing volatile organic compounds (VOCs).^20^ Thus, the resistance of tomato plants to herbivores is likely to be associated with the type and density of trichomes, and the efficient release of metabolites and volatiles, which both depend on the genetic background (i.e. cultivated variety or wild tomato).^21^ However, tomato breeding programmes have devoted strong attention to reducing genetic variability, and increasing fruit quality and quantity to meet consumer demands and improve market competitiveness, but little attention has been given to genes improving herbivore resistance.^22^ Therefore, tomato production systems have been facing serious problems with herbivorous insects including the tomato leaf miner *Phthorimaea absoluta* (formerly *Tuta absoluta*) (Meyrick) (Lepidoptera: Gelechiidae).^23^, ^24^

*Phthorimaea absoluta*, originating from South America, is one of the most devastating insects that threatens tomato production worldwide.^25^ The pest can damage any aerial part of tomato plants, including leaves, stems and fruit, and has recently been reported to horizontally transmit tomato brown rugose fruit virus to healthy tomato plants.^26^ Preferentially, the larvae feed on mesophyll tissues of the leaves, reducing their photosynthetic capacity and leading to a reduction in yield and fruit quality.^27^ Synthetic pesticides have been adopted as an emergency response but the concealed feeding mode of the larvae, high costs, the development of resistance, and negative impacts on the environment and human health make this method unsustainable.^28^, ^29^ Consequently, alternative approaches to the management of *P. absoluta* are exploited. Among those, using resistant plants remains an eco‐friendly approach and is considered an important component of integrated pest management (IPM).^29^ Although most commercial varieties possess weak defences against *P. absoluta*,^13^ differences in resistance to the pest have been observed.^30^ Those differences were linked to the density of trichomes and the diversity and concentration of secondary metabolites.^31^, ^32^, ^33^ There is still a need to develop domesticated tomato plants that exhibit better performance against the pest.^23^ Moreover, because wild relatives of tomatoes often exhibit high resistance to *P. absoluta* and are used in breeding commercial varieties,^33^ exploring wild tomato species for enhancing resistance in domesticated tomato through interspecific crosses remains highly relevant.^33^, ^34^ Thus, it is important to continue searching for tomato plants with diversified morphological and chemical traits that offer resistance to *P. absoluta*.

This study aims to evaluate the performance of 19 diversified genotypes of tomato, including cultivated varieties and wild species, against *P. absoluta* and understanding the role of plant trichomes in the resistance of tomato to the pest. To achieve this, we evaluated: (i) the oviposition response of female *P. absoluta* across tomato genotypes, (ii) the larval life‐history parameters on different tomato genotypes, and (iii) the types and density of trichomes on tomato plants.

## 2 MATERIAL AND METHODS

### 2.1 Plant material

Nineteen tomato genotypes comprising 16 commercial varieties and three wild species were selected for this study (Table 1). Wild tomatoes *Solanum arcanum* and *S. neorickii* were donated by the US Davis/CM Rick Tomato Genetic Resource Center, Davis, MC 95616 (TGRC). The other genotypes were purchased from commercial suppliers in Switzerland. The selected domesticated varieties are commonly grown in Switzerland and also known to exhibit resistance against common diseases (Table 1). The selection of wild tomato was based on the presence of various physical traits and also on the fact that they have shown resistance against lepidopteran insects including *P. absoluta*.^37^, ^38^ Before sowing, the wild species *S. arcanum* and *S. neorickii* were bleached by soaking seeds in 3% hypochlorite solution for 30 min as recommended by TGRC. Thereafter, bleached seeds were rinsed for several minutes under running water and sown immediately.^39^ Seeds of each genotype were grown in plastic transplant trays containing organic substrate for a month. Subsequently, seedlings were individually transplanted into pots (10‐cm diameter) containing organic substrate enriched with long‐term fertilizer (3 kg m^−3^, Manna Cote 4M; Hauert HBG Dünger AG, Grossaffoltern, Germany) and maintained in the glasshouse at 25 ± 5 °C and 60 ± 10% relative humidity (RH). Plants were supported with a string trellis attached to an overhead rope and watered three times a week with no addition of insecticides. Plants used in all experiments were ≈4–6 weeks post‐transplanting and ranged in height from 0.8 and 2 m. For the rearing of *P. absoluta*, the Rentita tomato variety at 4–8 weeks post‐transplanting was used. This variety was selected based on its plant architecture and suitability in terms of leaf production (preliminary observations).

**Table 1:** Tomato genotypes used in the experiments

Taxon	Genotypes	Characteristics	Source
*Solanum lycopersicum* L.	Starbuck F1	Resistant to ToMV, Ff, Va, Vd	Bigler Samen AG, Thoune, Switzerland
	Tomimaru Muchoo	Resistant to ToMV, Ff, Fol, For, Ma, Mi, Mj, Si	
	Admiro F1	Resistant to ToMV, Va, Vd, On, For	
	Cindel F1	Moderately resistant to *P. absoluta* ^35^ Resistant to ToMV, Fol, Va, Vd	
	Berner Rose	Very vigorous plant	
	Corona F1	Resistant to ToMV, Ff, Fol, Va, Vd, Ma, Mi, Mj, Ss	
	Aurea F1	Resistant to ToMV, Va, Vd	
	Romabelle F1	Resistant to ToMV, Va, Vd, Fol, For, Ma, Mi, Mj, On	
	Goldene Königin	Resistant to ToMV, Fol	
	Beorange F1	Vigorous plant, resistant to ToMV, Ff, Fol, For, Va, Vd	
	Indalo F1	Vigorous plant with dense foliage	
	Green Zebra	Vigorous plant	
	Costoluto Genovese F1	‐	
	Previa F1	Resistant to ToMV, Fol, Vd, For, On	
	Noire de Crimée	‐	
	Rentita	Variety utilized for the rearing of *P. absoluta*	
*S. pimpinellifolium* ‐	‐	Susceptible^36^ and partially resistant against *P. absoluta* ^17^	Samen Mauser, Winterthur, Switzerland
*S. arcanum* LA1031	‐	High density of glandular trichomes^37^	Tomato Genetic Resource Center (TGRC, Davis, USA)
*S. neorickii* LA1329	‐	Resistant against *P. absoluta* ^38^	

*Note*: ToMV, Tomato Mosaic Virus; Ff, *Fulvia fulva*; Va, *Verticillium albo*‐*atrum*; Vd, *Verticillium dahliae*; Fol, *Fusarium oxysporum* f. sp. *lycopersici*; For, *Fusarium oxysporum* f. *sp. Radicis licopersici*; Ma, *Meloidogyne arenaria*; Mi, *M. incognita*; Mj, *M. javanica*; Si, Silvering; Ss, Rhizomonas/*Sphingomonas suberifaciens*; On, *Oidium neolycopersici*.

### 2.2 Phthorimaea absoluta

The *P. absoluta* laboratory colony was established using ≈200 pupae provided by Andermatt Biocontrol (Grossdietwil, Switzerland). The initial collection was made in 2011 in Ticino, Switzerland, and has been refreshed regularly with individuals from the wild, the last time in 2022, shortly before our experiments. Pupae were placed in a mesh cage (50 × 50 × 50 cm; bug dorm; MegaView Science Co. Ltd., Taichung City, Taiwan), supplemented with drops of honey provided on a post‐it® paper. Upon adult emergence, cut tomato plants were introduced in the cage for infestation. The tomato plant stem was placed inside a 1‐L Erlenmeyer flask filled with water to keep the plant hydrated. After 72 h, infested plants were removed and kept in a new cage to commence a new colony. For larval development, fresh tomato plants were supplied until pupation.

In order to obtain newly emerged adult moths for the experiments, single cut tomato plants having their stem placed inside a 1‐L Erlenmeyer flask filled with water were exposed to ≈200 adults (ratio of approximate 1:1, male: female) in cages (50 × 50 × 50 cm) for oviposition over a 4‐h period. Thereafter, the infested plant was removed and placed in an empty cage (50 × 50 × 50 cm) for larval development. Fresh tomato plants were provided until the 4^th^‐instar larvae started pupating. Pupae were collected and sexed following an examination of the distinctive sexual dimorphism displayed in the final segments of the pupal abdomen^40^ using a stereomicroscope (×100, MZ125; Leica, Wetzlar, Germany), and 190 pupal pairs were individually set in a Petri dish (3 cm diameter) for emergence.

All insect rearing and experiments were performed in climate chambers at 25 ± 1 °C, 70 ± 10% RH and a 16 h:8 h, light:dark photoperiod.

### 2.3 Resistance assessment

#### 2.3.1 Oviposition response under nonchoice conditions

In order to evaluate the effect of tomato genotypes on *P. absoluta* oviposition, leaves from the middle part of each tomato (3–5 leaflets) were detached from potted plants and introduced in 1.3‐L plastic jars covered with a fine mesh. These leaves are fully expanded and offer consistent maturity, optimal nutrient levels and balanced trichome density, making them suitable for resistance evaluation.^17^, ^41^, ^42^ The leaf petiole extending from the jar through a hole was submerged into a water‐filled container to keep the plant fresh. One pair of newly emerged *P. absoluta* (<24 h) was released in the test arena where the female was allowed to lay eggs for 48 h. After this period, leaves were removed and new healthy leaves were placed in the jars until the female died. When one adult died, two more leaf renewals were performed. If no eggs were laid during this period, we assumed that the female died and the experiment ended, whereas if infestation was noticed, a male the same age as the female was introduced. Removed leaves were checked and the number of eggs on the lower and upper side of each leaf was recorded. This experiment was replicated 10 times.

#### 2.3.2 Egg‐hatching assay

This experiment aimed to evaluate whether tomato trichomes affect the duration of the egg stage of *P. absoluta*, based on reports that a plant's physical traits can damage pest eggs and potentially influence their hatching.^43^ Tomato leaves of each genotype were provided to pairs of newly emerged *P. absoluta* for oviposition as described above. Before the experiment, the pairs were individually kept in a Petri dish (as described above) for 48 h to ensure that mating occurred. Each tomato leaf contained a single leaflet (all other leaflets were removed with a scissor). After 24 h, moths were removed and 10 eggs were randomly marked with a circle on the leaf using a permanent marker. Thereafter, the duration of the egg stage (referring to hatching period) and number of hatched eggs was recorded daily. The eggs were considered hatched when the embryo inside successfully emerged from the eggshell, as confirmed by observing the newly hatched larvae. This experiment was replicated 10 times per genotype.

#### 2.3.3 Larval performance under nonchoice conditions

In order to obtain 1^st^‐instar *P. absoluta* larvae for the feeding experiment, eggs were produced by releasing adult moths of mixed sexes (>100; 24 h old) in 1.3‐L plastic jars covered with fine mesh. Tomato leaves (var. Rentita) were placed on top of the mesh to provide an odour source. Female moths were allowed to lay eggs on the bottom of the mesh overnight for ≈16 h. After this period, moths were removed and eggs hatched ≈72 h postoviposition.

Tomato leaflets from the middle part of each plant were cut and the petioles of leaflets were wrapped in wet cotton to keep them hydrated. The leaflets were kept in plastic boxes (12.5 cm length, 10 cm width, 5 cm height) covered with fine mesh. A single 1^st^‐instar larva was released on each leaflet using a fine brush; no additional leaflet was supplied for larvae during their development. Upon pupation, the pupae were individually collected, sexed under a stereomicroscope (as described above), weighed using a sensitive balance (DeltaRange AT261; Mettler‐Toledo International Inc., Columbus, OH, USA), and placed in Petri dishes (3 cm diameter). The emergence of adult moths was monitored daily. Data were collected on the time taken for larva to complete leaflet penetration (larval settlement time), larval development time, larval survival, pupal development time and pupation rate. Survival was assessed daily until pupation. The larval settlement time was recorded through visual observation at 20‐min intervals for each leaflet, from the moment of larval release until full penetration into the leaflet. The feeding damage inflicted by larvae was assessed by taking a picture of each leaflet and estimating the consumed area using the program imagej v1.48.^44^ This experiment was replicated 30 times per genotype.

### 2.4 Trichome assessment

Trichome types and densities were assessed on the upper and lower leaf side for each tomato genotype. The categorization of trichome types followed the description provided by Bar *et al*.^45^ Measurements were taken on eight leaflets from eight different potted plants (4–6 weeks old post‐transplanting). Leaflets were chosen from leaves of the middle part of the plants and a 2 cm^2^ section was cut and placed on a glass slide. Counts were made using three sections (apex, margins and edges) from each leaflet using a digital stereo‐microscope (VHX 6000; Keyence, Osaka, Japan). For all genotypes except *S. arcanum*, the sampling units were an area of 4 mm^2^ for all types of glandular trichomes and for all types of nonglandular trichomes on the upper side of the leaflet as well as for nonglandular types II and III on the lower side of the leaflet. For nonglandular trichomes type V and VIII on the lower side of the leaflet, the sampling unit was an area of 1 mm^2^. For *S. arcanum*, the sampling unit was an area of 4 mm^2^ for glandular trichomes VI and VII on both sides of the leaflet, and 1 mm^2^ for glandular trichomes I and IV on both sides of the leaflets. Differences in sampling units were to the consequence of a high density of nonglandular trichomes observed on the lower side of all leaflets and glandular trichomes of *S. arcanum*. To standardize trichome density in the data analysis, the trichome densities assessed at 1 mm^2^ were quadrupled and the sum of the three sections from each side of the leaflet, totalling a surface area of 24 mm^2^ per leaflet, was considered.

### 2.5 Data analysis

All data were checked for normality and homogeneity of variance using Shapiro–Wilk and Levene's tests, respectively. One‐way ANOVA was performed to analyze the effect of tomato genotypes on female total oviposition and female pupal weight. In instances where significant differences were observed, Tukey's honestly significant difference test (*P* < 0.05) was employed for *post hoc* mean separation. The pupation rate, adult emergence, adults with deformities and total number individual negatively affected were calculated using the following formula:

$$\text{Pupation rate}=\frac{\text{Number of pupated larvae}\;}{\text{Total number of larvae}}\;\times \;100$$

$$\text{Adult emergence}=\frac{\text{Number of emerged pupae}}{\text{Total number of pupae}}\;\times \;100$$

$$\text{Adults with deformities}=\frac{\text{Number of adults with deformities}}{\text{Total number of emerged adults}}\;\times \;100$$

$$\begin{array}{l} \text{Total number of individual negatively affected}\\ \;=\begin{array}{l} \frac{\mathrm{No}.\;\text{of nonpupating larvae}+\mathrm{No}.\;\text{of nonemerged larvae}+\mathrm{No}.\;\text{of adults with deformities}}{\text{Total number of larvae}}\;\times \;100 \end{array} \end{array}$$

Because the data did not exhibit overdispersion, the effect of tomato genotypes on egg hatching and larval survival were modeled using binomial generalized linear models (GLM) with a logit link function. To investigate the relationship between oviposition and timing of oviposition across genotypes, we employed nonlinear least squares (NLS) regression analysis using the function ‘nls’. We fitted the following exponential growth model to the data:

$$Y=a\;\times \;\exp\;\left(r\;\times \;X\right).$$

where *Y* is oviposition (no. of eggs laid) and *X* is oviposition period (days).

The model was fitted using NLS regression, initialized with starting values *a* = 50 and *b* = −0.04. The model estimated parameters *a* and *r* were 100.31 (SE = 3.05, *t*‐value = 32.87, *P* < 0.001) and −0.251 (SE = 0.0085, *t*‐value = −29.67, *P* < 0.001), respectively, and residual SE was 14.23, indicating a good fit of the model to the data.

The correlations between number of eggs and oviposition periods were assessed using Spearman rank correlation. A nonparametric Kruskal–Wallis test was conducted to assess the effect of tomato genotypes on egg hatching period, larval settlement time, larval period, pupal period, damaged leaflets area and male pupal weight, as well as density of trichomes of tomato genotypes. Dunn's test was applied for multiple comparisons among genotypes. The multivariate method of principal component analysis (PCA) was performed using the function ‘prcomp’ to explore the relationships between trichome density and female oviposition, and between trichome density and larval settlement time. Additionally, the analysis examined the correlation between damaged area and pupal weight across all tomato genotypes. All analyses were performed using R software v4.4.1.^46^

## 3 RESULTS

### 3.1 Oviposition assay

**Figure 1:**
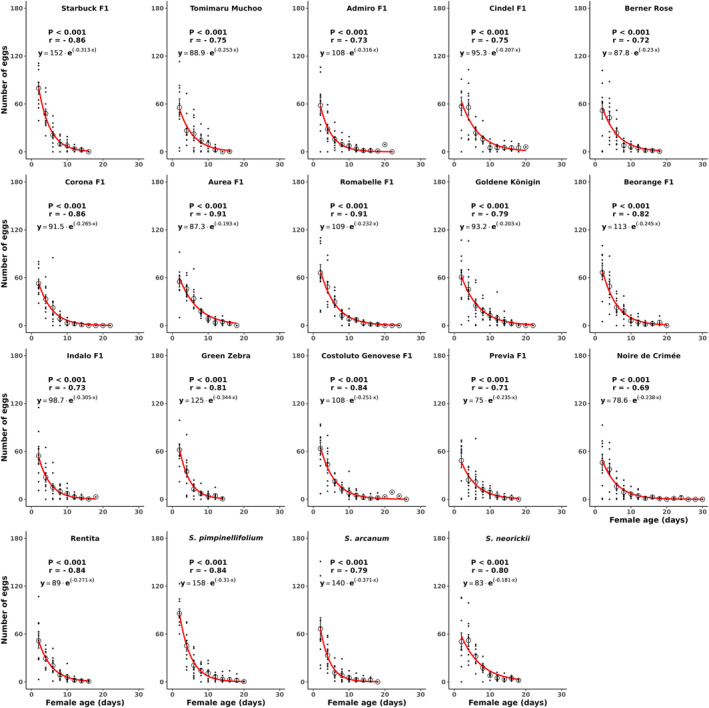
Daily oviposition (no. of eggs laid) of *Phthorimaea absoluta* across 19 tomato genotypes. The interaction between oviposition and days was assessed by computing a simple exponential curve [*Y* = *a* × exp (*r* × *X*)] and Spearman's correlation coefficient (*r*) (<0.001). *n* = 10.

The total oviposition of female *P. absoluta* differed significantly among the tested tomato genotypes (*F* = 2.31, df = 18, *P* = 0.002) (Table 2). Females laid eggs on all genotypes and total oviposition per female ranged from 114 to 186 eggs. The largest number of eggs was found on the wild species *S. pimpinellifolium*, followed by the variety Goldene Königin. The lowest total oviposition was recorded on variety Rentita followed by Corona F1, Indalo F1, Noire de Crimée and Previa F1 (Table 2). Furthermore, daily oviposition was significantly affected by the factors genotype (*F* = 3.6415, df = 18, *P* < 0.001) and female age (*F* = 2931.34, df = 1, *P* < 0.001). The interaction between genotype and female age also was significant (*F* = 1.9295, df = 18, *P* = 0.01). Oviposition was negatively correlated with days across all genotypes (Fig. 1). Tomato genotype did not delay egg laying by female *P. absoluta*, as all moth females started to lay eggs within the first 2 days (Fig. 1), but oviposition was higher on *S. pimpinellifolium* and the variety Starbuck compared to other genotypes during that period (*F* = 2.15, df = 18, *P* = 0.005). For all genotypes, >79% of eggs were laid during the first 6 days except for Goldene Königin (73.81%).

**Table 2:** Density of glandular, nonglandular trichomes and total trichomes (number of trichomes/24 mm^2^/leaflet), mean number of eggs and larval settlement time by *Phthorimaea absoluta*. All data are presented as mean ± SE.

Genotypes	Density of glandular trichomes per 24 mm^2^ (*n* ^†^ = 8)	Density of nonglandular trichomes per 24 mm^2^ (*n* ^†^ = 8)	Total trichomes per 24 mm^2^ (*n* ^†^ = 8)	Mean number of eggs per plant (*n* ^‡^ = 10)	Larval settlement time (h) (*n* ^§^ = 30)
*Solanum lycopersicum*					
Starbuck F1	26.7 ± 6.87 i	303.6 ± 13.08 m	330.4 ± 12.57 k	167.0 ± 18.44 c	1.0 ± 0.04 de
Tomimaru Muchoo	32.0 ± 11.01 h	307.9 ± 28.32 m	339.9 ± 31.03 j	127.4 ± 21.3 f	1.2 ± 0.07 cd
Admiro F1	55.0 ± 7.95 d	426.9 ± 65.13 f	481.9 ± 68.69 f	120.8 ± 17.6 g	1.1 ± 0.07 cde
Cindel F1	9.1 ± 1.77 k	257.9 ± 22.76 o	267.0 ± 21.87 L	159.2 ± 21.92 d	1.0 ± 0.04 de
Berner Rose	41.5 ± 6.97 fg	388.9 ± 24.29 i	430.4 ± 30.00 h	134.9 ± 16.63 e	1.0 ± 0.03 de
Corona F1	63.9 ± 9.97 c	509.0 ± 88.37 c	572.9 ± 83.95 d	115.6 ± 15.92 gh	0.8 ± 0.06 e
Aurea F1	65.6 ± 7.16 c	549.0 ± 30.69 b	614.6 ± 34.64 b	168.2 ± 9.5 c	1.1 ± 0.05 cde
Romabelle F1	18.0 ± 3.41 i	369.1 ± 24.55 j	387.1 ± 22.90 i	171.7 ± 15.15 c	1.1 ± 0.11 cde
Goldene Königin	23.7 ± 8.27 i	407.9 ± 27.47 g	431.6 ± 31.10 h	177.2 ± 18.81 b	1.3 ± 0.08 bcd
Beorange F1	63.2 ± 12.7 c	323.0 ± 28.92 L	386.2 ± 36.10 i	170.8 ± 20.59 c	1.0 ± 0.08 de
Indalo F1	46.2 ± 11.49 e	452.9 ± 48.56e	499.1 ± 57.55 e	116.0 ± 19.12 gh	1.0 ± 0.07 de
Green Zebra	32.4 ± 8.21 h	292.7 ± 16.95 n	325.1 ± 19.74 k	120.8 ± 10.78 g	1.4 ± 0.07 bc
Costoluto Gen F1	32.9 ± 4.71 h	213.7 ± 25.56 *P*	246.6 ± 25.16 m	159.0 ± 13.02 d	1.0 ± 0.05 de
Previa F1	39.5 ± 5.83 g	459.2 ± 53.56 d	498.7 ± 56.18 e	118.8 ± 11.03 gh	1.1 ± 0.04 cde
Noire de Crimée	62.9 ± 16.20 c	505.2 ± 56.06 c	568.1 ± 70.59 d	118.3 ± 22.93 gh	1.2 ± 0.04 cd
Rentita	74.2 ± 11.22 b	356.9 ± 85.32 k	431.1 ± 93.11 h	114.2 ± 13.98 h	1.5 ± 0.08 b
*S. pimpinellifolium*	33.4 ± 6.40 h	563.1 ± 75.66 a	596.5 ± 81.21 c	186.6 ± 9.58 a	1.1 ± 0.07 cde
*S. arcanum*	780.4 ± 45.02 a	0.0 ± 0.0 q	780.3 ± 45.02 a	129.4 ± 16.29 f	1.9 ± 0.17 a
*S. neorickii*	44.6 ± 6.47 ef	396.3 ± 23.05 h	441.0 ± 24.89 g	166.9 ± 19.22 c	1.3 ± 0.07 bcd

*Note*: Means followed by different letters within a column are significantly different (total oviposition: ANOVA, *P* < 0.01, followed by Tukey's honestly significant difference *post hoc* test; trichome density and settlement time: Kruskal–Wallis test, *P* < 0.001, followed by Dunn's test).

† Number of leaflets from different tomato plants of the same genotype.

‡ Number of female *P. absoluta* per genotype.

§ Number of larvae per genotype.

### 3.2 Egg hatching

**Figure 2:**
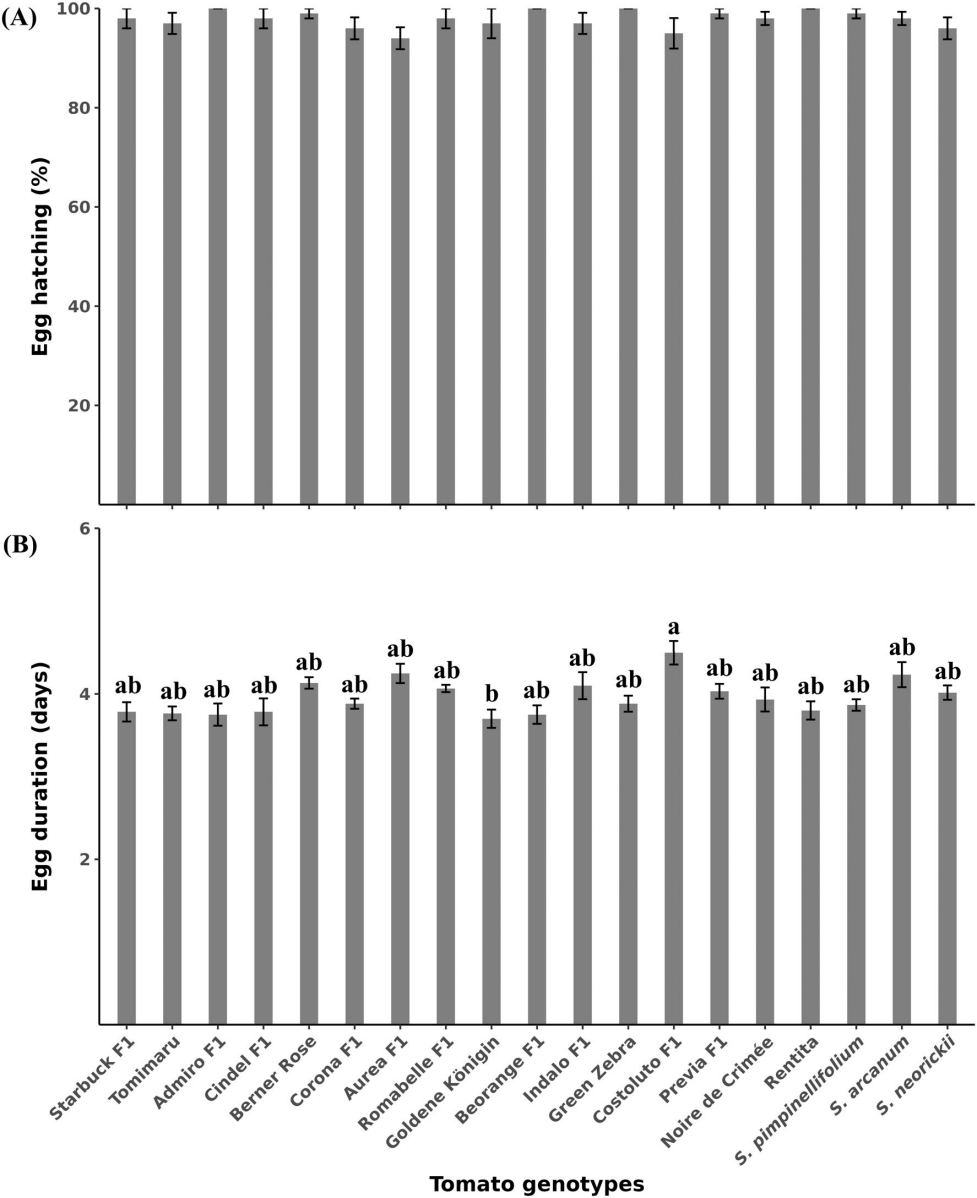
Percentage of egg hatching (A) and duration of egg stage (B) of *Phthorimaea absoluta* on 19 tomato genotypes. Tomimaru Muchoo (Tomimaru) and Costoluto Genovese F1 (Costoluto F1). Means (±SE) with different letters are significantly different (A: GLMs with binomial distribution and log‐link function, *P* > 0.05; B: Kruskal–Wallis test, followed by Dunn's test, *P* < 0.001). *n* = 10.

The percentage of hatched eggs varied between 95% and 100% and did not differ among tomato genotypes (*χ* ^2^ = 3.0904, df = 18, *P* > 0.05) [Fig. 2(A)]. However, the duration of egg stage was significantly affected by genotype (*χ* ^2^ = 51.405, df = 18, *P* < 0.001) [Fig. 2(B)]. The longest duration until neonate emergence was on Costoluto Genovese F1 (4.5 ± 0.1 days) and the shortest on Goldene Königin (3.7 ± 0.1 days) [Fig. 2(B)].

### 3.3 Feeding assay

**Figure 3:**
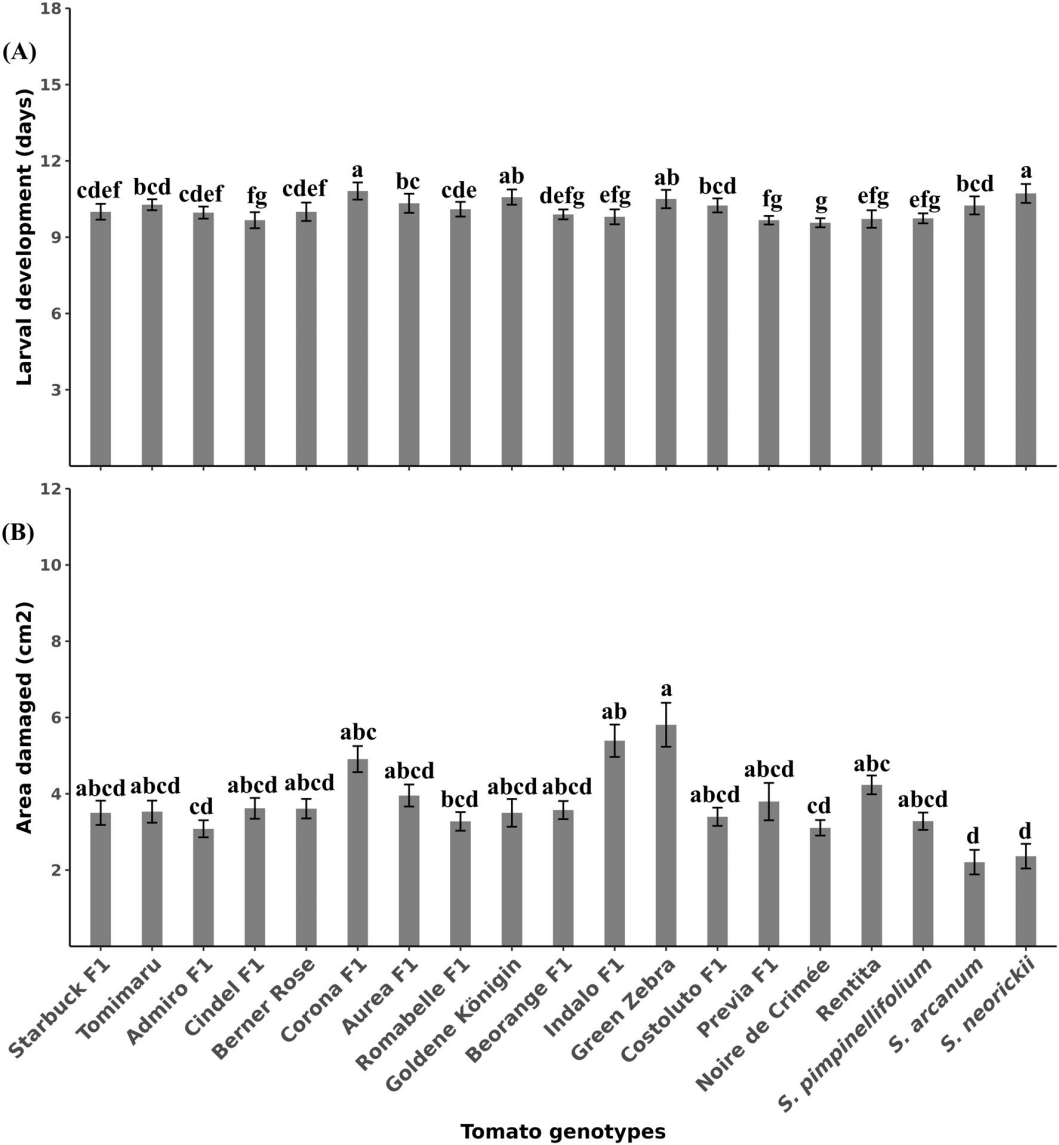
Larval development of *Phthorimaea absoluta* (A) and (B) leaflet area damaged on 19 tomato genotypes. Tomimaru Muchoo (Tomimaru) and Costoluto Genovese F1 (Costoluto F1). Damaged area was measured by taking a picture of each leaflet and estimating the consumed area using the program imagej v1.48. Means (±SE) with different letters are significantly different (Kruskal–Wallis test, followed by Dunn's test A: *P* < 0.05, *n* = 30; B: *P* < 0.001, *n* = 15).

**Figure 4:**
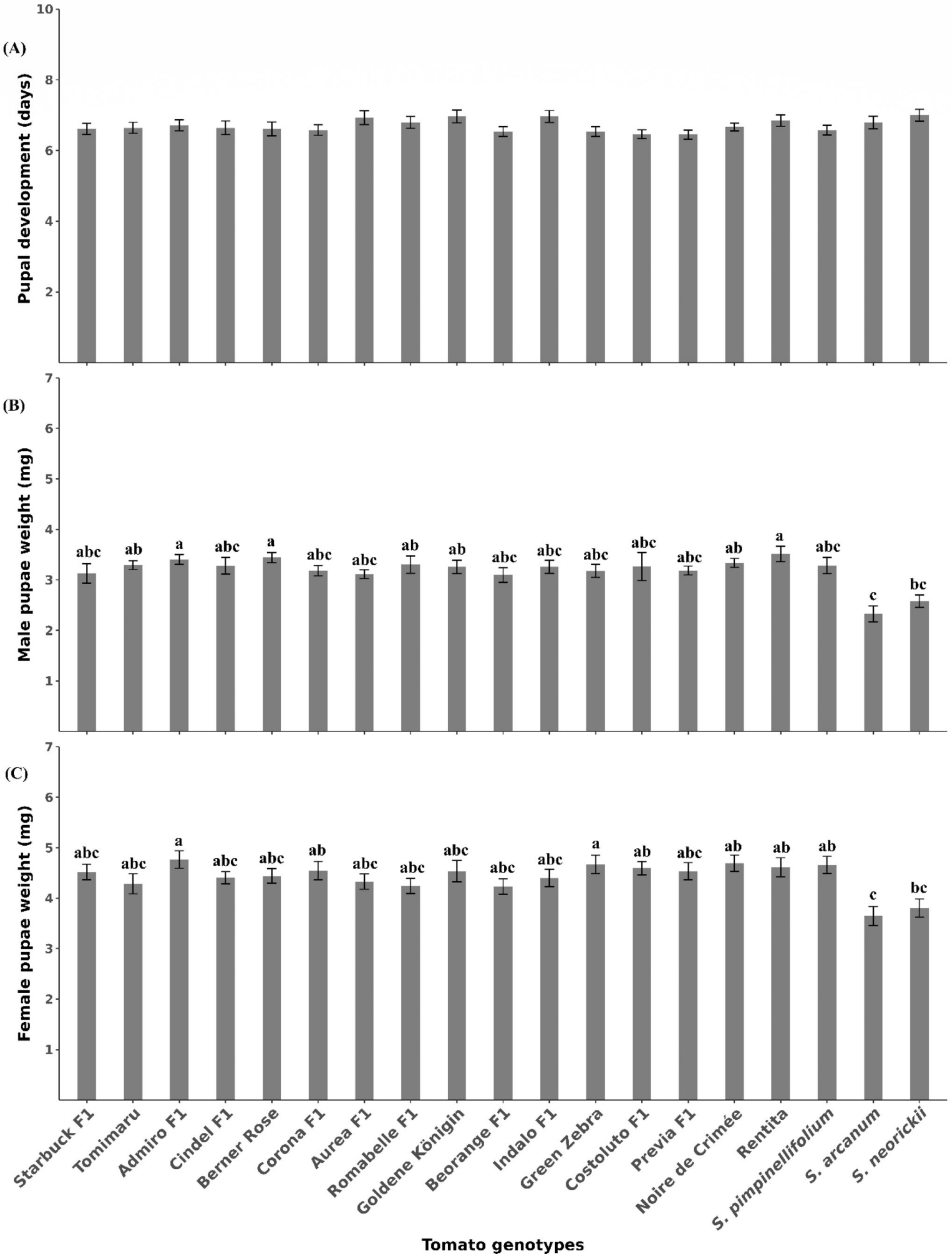
Pupal development (A), male pupal weight (B) and female pupal weight (C). Tomimaru Muchoo (Tomimaru) and Costoluto Genovese F1 (Costoluto F1). Means (±SE) with different letters are significantly (A: Kruskal–Wallis test, *P* > 0.05; B: Kruskal–Wallis test, followed by Dunn's test, *P* < 0.001; C: ANOVA, followed by Tukey's honestly significant difference *post hoc* test, *P* < 0.001). *n* = 30.

**Figure 5:**
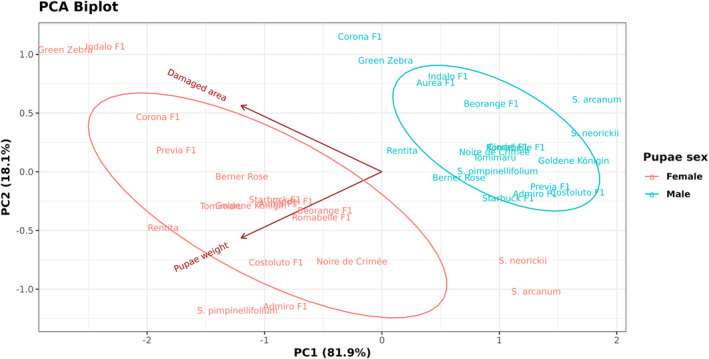
Principal component analysis (PCA) of damaged leaflet area and male and female pupal weight of *Phthorimaea absoluta* measured across 19 tomato genotypes. The distribution of the tomato genotypes is based on trichome density, total oviposition and larval settlement time of *P. absoluta*. The first principal component (PC1) explains 81.9% of the variance, and the second principal component (PC2) explains 18.1% of the variance. Colours indicate pupal sex and for each sex, closer genotypes produced similar pupal weights.

First‐instar larvae succeeded in settling in all tomato genotypes (Table 3) but the larval settlement time was significantly affected by tomato genotype (*χ* ^2^ = 120.64, df = 18, *P* < 0.001) (Table 2). The longest settlement time was on *S. arcanum* followed by Rentita (Table 2). The shortest time for settlement was observed on Corona F1. Moreover, larval development time significantly differed among genotypes (*χ* ^2^ = 30.322, df = 18, *P* = 0.03443) [Fig. 3(A)]. *Solanum neorickii* and Corona F1 exhibited the longest larval period and Noire de Crimée had the shortest [Fig. 3(A)]. Larval survival was not significantly affected by tomato genotype (*χ* ^2^ = 26.344, df = 18, *P* = 0.09215) even though the development of ≈20% of larvae on *S. arcanum*, *S. neorickii* and Corona F1 was negatively impaired (Table 3). Pupal development time did not differ among tomato genotypes (*χ* ^2^ = 22.393, df = 18, *P* = 0.215) [Fig. 4(A)]. Area of leaflets damaged by the larvae significantly depended on tomato genotype (*χ* ^2^ = 79.411, df = 18, *P* < 0.001) [Fig. 3(B)]. *Solanum arcanum* (2.2 ± 0.3 cm^2^) and *S. neorickii* (2.4 ± 0.3 cm^2^) were the least affected, whereas the highest damage was inflicted on Green Zebra (5.8 ± 0.6 cm^2^) and Indalo F1 (5.4 ± 0.4 cm^2^) [Fig. 4(A)]. Moreover, tomato genotype had a significant effect on the pupal weight of males (χ^2^ = 51.011, df = 18, *P* < 0.001) [Fig. 4(B)] and weight of females (*F* = 2.636, df = 18, *P* < 0.001) [Fig. 4(C)]. *Solanum arcanum* and *S. neorickii* yielded the lightest male and female pupae, respectively [Figs 4(B),(C) and 5]. The heaviest female pupae were observed on Admiro F1 and Green Zebra [Figs 3(C) and 5], whereas Rentita, Berner Rose and Admiro F1 yielded the heaviest male pupae [Fig. 4(B) and 5]. There was a significant correlation between pupal weight and damaged area for both sexes (Fig. 5).

**Table 3:** Percentage of larval settlement, pupation, adult emergence, adults with deformities and total number of individuals negatively impacted

Genotype	Settlement (%)	Pupation (%)	Adult emergence (%)	Deformities (%)	Total number of individuals negatively impacted (%)
*Solanum lycopersicum*					
Starbuck F1	100 (30)	100 (30)	93.33 (28)	3.35 (01)	10 (3)
Tomimaru Muchoo	100 (30)	96.66 (29)	96.55 (28)	0	6.66 (2)
Admiro F1	100 (30)	100 (30)	93.33 (28)	3.35 (01)	10 (3)
Cindel F1	100 (30)	100 (30)	93.33 (28)	7.14 (02)	13.33 (4)
Berner Rose	100 (30)	96.66 (29)	96.55 (28)	0	6.66 (2)
Corona F1	100 (30)	90 (27)	96.29 (26)	7.69 (02)	20 (6)
Aurea F1	100 (30)	100 (30)	93.33 (28)	3.57 (01)	10 (3)
Romabelle F1	100 (30)	100 (30)	96.66 (29)	0	3.33 (1)
Goldene Königin	100 (30)	86.66 (26)	100 (26)	3.84 (01)	16.66 (5)
Beorange F1	100 (30)	96.66 (29)	100 (28)	3.57 (01)	10 (3)
Indalo F1	100 (30)	100 (30)	96.66 (29)	0	3.33 (1)
Green Zebra	100 (30)	86.66 (26)	100 (26)	0	13.33 (4)
Costoluto Genovese F1	100 (30)	93.33 (28)	100 (28)	3.57 (01)	10 (3)
Previa F1	100 (30)	100 (30)	96.66 (29)	0	3.33 (1)
Noire de Crimée	100 (30)	100 (30)	100 (30)	0	0
Rentita	100 (30)	93.33 (28)	92.3 (24)	0	13.33 (4)
*S. pimpinellifolium*	100 (30)	90 (27)	96.29 (26)	3.84 (01)	16.66 (5)
*S. arcanum*	100 (30)	93.33 (28)	85.71 (24)	0	20 (6)
*S. neorickii*	100 (30)	83.33 (25)	96 (24)	4 (01)	23.33 (7)

*Note*: Sample size *n* is given in parentheses.

### 3.4 Trichome density

Glandular and nonglandular trichomes were observed on all tomato genotypes, except for *S. arcanum* which lacked nonglandular trichomes (Table 2). Total trichome density differed among genotypes (*χ* ^2^ = 79.109, df = 18, *P* < 0.001) (Table 2). Trichomes were most abundant on *S. arcanum* followed by Aurea F1 and *S. pimpinellifolium*, and the least abundant on Costoluto Genovese F1 and Cindel F1. Moreover, the total density of glandular and nonglandular trichomes varied by genotype (glandular: *χ* ^2^ = 80.103, df = 18, *P* < 0.001; nonglandular: *χ* ^2^ = 81.135, df = 18, *P* < 0.001) (Table 2). The highest density of glandular trichomes was on *S. arcanum* followed by Rentita and the lowest on Cindel F1 and Romabelle F1. Moreover, *S. pimpinellifolium* and Aurea F1 showed the most abundant nonglandular trichomes, whereas Green Zebra, Cindel F1 and Costoluto Genovese F1 presented the lowest (Table 2). Furthermore, *S. arcanum* was dominated by glandular trichomes types I and IV, whereas the other genotypes were dominated by nonglandular trichomes V and VIII (Data S1).

### 3.5 Relation between trichome density, oviposition and larval settlement time

**Figure 6:**
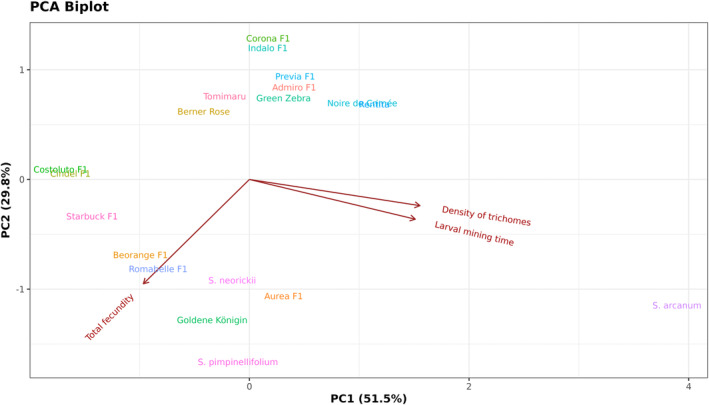
Principal component analysis (PCA) of trichome density, total oviposition and larval settlement time of *Phthorimaea absoluta* measured across 19 tomato genotypes. A two‐dimensional PCA plot showing the distribution of the tomato genotypes based on trichome density, total oviposition, and larval settlement time of *P. absoluta*. The first principal component (PC1) explains 51.5% of the variance, and the second principal component (PC2) explains 29.8% of the variance. Vectors pointing to the same direction are strongly correlated. Closer genotypes exhibit similar effects on the pest.

The first two PCA components explained 81% of variability (Fig. 5). The total number of trichomes was strongly positively correlated with larval settlement time, whereas total oviposition and total number of trichomes were weakly correlated. Moreover, the higher the trichome density, the longer the larval settlement on the leaflets (Fig. 6), especially when considering glandular trichome types I and IV abundantly present on the wild species *S. arcanum* (Data S1).

## 4 DISCUSSION

Our study revealed distinct differences in susceptibility to *P. absoluta* attacks and variations in trichome density among tomato genotypes. While trichome density did not influence female oviposition preference, it played an important role for larval establishment on tomato leaves. Interestingly, resistance through antixenosis was present in several domesticated tomatoes and in the wild species *S. arcanum*, whereas resistance through antibiosis was found in two wild species (*S. arcanum* and *S. neorickii*) as well as in the domesticated tomato Corona F1, demonstrating that host plant resistance can be a promising tool in managing this invasive species. This study therefore complements the range of studies reporting on different aspects of tomato resistance (Table 4) by a thorough evaluation of potentially affected parameters in the *P. absoluta* life‐cycle. It has to be noted, however, that our study was conducted under contained conditions with detached tomato leaves. It thus did not consider the full range of host plant identification and selection behaviour of the adult moths.

**Table 4:** Antixenosis and antibiosis effects of tomato (*Solanum*) species on *Phthorimaea absoluta* as reported in the literature

References	Tomato species (variety, accession)	Antixenosis†		Antibiosis‡	
		No‐choice test	Choice test	No‐choice test	Choice test
47	*S. cheesmaniae* (VI037240–7) *S. galapagense* (VI057400‐3, VI063177‐10)	No	Yes	Yes	‐
	*S. lycopersicum* (Hawaii‐7996, CLN‐5915)	No	No	No	‐
19	*S. pennellii* (LA0716)	Yes	Yes	Yes/no	‐
	*S. habrochaites* (PI134417)	No	Yes	Yes	‐
	*S. lycopersicum* (Santa Clara)	No	No	No	‐
48	*S. habrochaites* (LA1777) *S. lycopersicum* (EC 620343)	Yes	‐	Yes	‐
	*S. lycopersicum* (EC620370, EC631369, EC705464, EC519819)	No	‐	No	‐
31	*S. chmielewskii* (LA1327) *S. lycopersicum* (CGN14330)	No	No	No	‐
	*S. chmielewskii* (LA1028),*S. galapagense* (LA0317),*S. habrochaites* (AusTRCf 312 070 (W—C1311)),*S. chilense* (LA1967) *S. lycopersicum* var. *cerasiforme* (LA1221)	Yes	‐	Yes	‐
	*S. chmielewskii* (LA1028), *S. galapagense* (LA0317)	‐	Yes	‐	‐
41	*S. lycopersicum* (Santa Clara, Moneymaker, TOM‐601)	‐	No		
	*S. esculentum* (HGB‐674, HGB‐1497)	‐	Yes	‐	‐
49	*S. lycopersicum* (Raha, Quintini, ES9090F1)	‐	Yes	‐	‐
	*S. lycopersicum* (Caroon, Iden, Firinze, Comodoro, Sunsid‐6189, GS‐12, Samar‐625)	‐	No	‐	‐
50	*S. habrochaites* (LA1777, LA1718, G1.1561) *S. pennellii* (LA716)	Yes	Yes	Yes	‐
	*S. lycopersicum* (Moneymaker)	‐	No	‐	‐
51	*S. pennellii* (LA716) *S. habrochaites* (PI127826)	‐	Yes	‐	Yes
52	*S. lycopersicum* Berlina, Zaman, and Golsar	‐	Yes	‐	Yes
	*S. lycopersicum* (Ps‐6515, Poolad, Petoprid‐5, Matin, Sandokan‐F1, Golshan‐616, Sadeen‐95, Sadeen‐21)	‐	No	‐	No
32	*S. hirsutum* f. *glabratum* (PI134417)	Yes		Yes	
	*S. esculentum* (Santa Clara)	No	‐	No	‐
53	*S. lycopersicum* (BR221)	‐	Yes	‐	‐
	*S. lycopersicum* (PS650)	‐	No	‐	‐
54	*S. hirsutum* (Peto Mech, Río Grande, King Ston)	‐	Yes	‐	Yes
	*S. hirsutum* (Mobil, Falat 3, Cal J N3, Dehghan, Super Strain B, Early Urbana)	‐	No	‐	No
17	*S. habrochaites* (RCAT030597, PI126446) *S. chilense* (INIA BB79) *S. peruvianum* (RCAT031296, RCAT039874, RCAT030403) *S. pimpinellifolium* (PI390739)	‐	Yes	‐	‐
	*S. habrochaites* (RCAT030597) *S. peruvianum* (RCAT039874)	‐	‐	Yes	‐
55	*S. lycopersicum* (Early Urbana Y, Primo Early)	Yes	‐	Yes	‐
	*S. lycopersicum* (Rio Grande, Cal JN3, Petomech, Super Strain B, Super 2270)	No	‐	No	‐
30	*S. lycopersicum* (Korral, CH Falat)	‐	Yes	Yes	‐
	*S. lycopersicum* (Chef, Infinity, Pellmech, Cal JN3, Valouro)	‐	No	No	‐
56	*S. galapagense* (VI037241) *S. cheesmaniae* (VI037240) *S. habrochaites* (LA1777, *glabratum* VI030462)	‐	Yes	Yes	Yes
57	*S. lycopersicum* (Early Urbana, Rio grande)	‐	‐	‐	Yes
	*S. lycopersicum* (Primo early, Calj N3, Peto mek, Supper 2270)	‐	‐	‐	No
38	*S. pennellii* *S. neorickii* *S. lycopersicum* (Depar)	Yes	‐	‐	‐
	*S. chilense* *S. corneliomulleri*	No	‐	‐	‐
	*S. corneliomulleri* *S. neorickii* *S. lycopersicum* cv Depar	‐	‐	Yes	‐
34	*S. chmielewskii* (LA1036) *S. habrochaites* (*hirsutum* PI127826, *glabratum* PI247087) *S. pennellii* (LA716)	‐	Yes		
	*S. pennellii* (LA716) *S. habrochaites* (*hirsutum* PI127826) *S. chmielewskii* (LA10136) *S. galapagense* (LA1401)	‐	‐	‐	Yes
	*S. lycopersicum* (Redenção, *cerasiforme*) *S. peruvianum* (LA0153)	‐	No	‐	No
58	*S. lycopersicum* (Patio Princess, Qualit 23)	No	No	No	‐
36	*S. cheesmaniae* (VI037240) *S. galapagense* (VI063177)	Yes	Yes	Yes	‐
	*S. pimpinellifolium* (VI030462) *S. lycopersicum* (CL5915)	No	No	No	‐

† Parameters such as number of eggs per plant/leaves or density of larvae per plant/leaves were assessed.

‡ Parameters such as larval mortality, larval period, damaged area, pupal period, pupal and adult weight, or adults emergence were assessed.

Our findings revealed that across all genotypes, *P. absoluta* laid the lowest number of eggs on several of the domesticated varieties. This indicates an antixenosis mechanism in domesticated tomatoes as has been reported before (Table 4). In addition to low oviposition, reduced larval survival and a prolonged larval development period was observed on the domesticated tomato Corona F1 in our study. This suggests not only an antixenosis, but also an antibiosis mechanism in this variety, making it partially resistant against *P. absoluta*. So far, reports of the antibiosis mechanism in domesticated tomato plants are limited to few studies.^30^, ^54^ Therefore, the domesticated tomato Corona F1 could be considered a component of IPM strategies, complementing other alternative methods to minimize the reliance on chemical insecticides.

Among the wild species tested, *S. pimpinellifolium* received the highest number of eggs, significantly more than the other two wild species, *S. arcanum* and *S. neorickii*. These species belong to distinct subsections within *Solanum* plants possessing distinct genetic diversity, with *S. pimpinellifolium* in the subsection Lycopersicon, and *S. arcanum* and *S. neorickii* in the subsection Arcanum.^59^ The increased oviposition of *P. absoluta* on *S. pimpinellifolium* suggests that this species may lack certain resistance traits found in species from the Arcanum subsection. An alternative explanation for the increased oviposition by *P. absoluta* on *S. pimpinellifolium* could be the level of constitutive volatiles, which can enhance a plant's suitability for infestation.^60^ However, the use of detached leaves in our study may have altered or reduced constitutive volatile emissions, potentially affecting the oviposition behaviour of *P. absoluta*. For instance, a higher level of methyl ketones and terpenes found constitutively in the wild tomato *S. habrochaites* (subsection Eriopersicon) exposed the plant to greater *P. absoluta* oviposition compared to the wild tomato *S. pennellii* (subsection Neolycopersicon).^19^ Moreover, higher oviposition of female *P. absoluta* also was reported on the wild tomatoes *S. chilense* (subsection Eriopersicon)^38^ and *S. cheesmaniae* LA1139 (subsection Lycopersicon).^50^ Therefore, breeding and selection strategies should account for these differences both within and across *Solanum* subsections to develop *P. absoluta*‐resistant tomato varieties. In contrast to our study, Kumaraswamy *et al*.^47^ reported similar oviposition across tomato genotypes, including both wild and domesticated tomatoes, under no‐choice conditions. The authors argued that in the absence of their preferred host, *P. absoluta* will lay eggs on any available host. This argument may also explain why there was no consistent correlation between adult oviposition and larval performance on the different genotypes in our experiments, as would be suggested by the preference–performance hypothesis.^41^ While female *P. absoluta* laid abundant eggs on tomato leaves that supported larval development and offspring fitness well, and in some cases (Corona F1 and *S. arcanum*) laid relatively few eggs on leaves that provided low support for larval growth and offspring fitness, abundant eggs also were laid on leaves that were less suitable for their offspring (e.g. *S. neorickii*).

We found that *P. absoluta* larvae took more time to settle on *S. arcanum*, the tomato genotype with the highest abundance of glandular trichomes, suggesting an antixenosis mechanism. Likewise, Guruswamy *et al*.^50^ reported that the wild tomato *S. habrochaites* LA 1777 exhibited such an antixenosis defence owing to greater abundance of glandular trichomes.^61^ Moreover, we found that on *S. arcanum* and *S. neorickii*, *P. absoluta* offspring development was adversely affected, visible as longer larval development time on *S. neorickii*, and reduced pupal weight and survival on both of these tomato species, which points to an antibiosis effect. Likewise, antibiosis effects of wild tomato genotypes, including *S. neorickii*, on *P. absoluta* were reported, such as compromised ability of larvae to achieve maturation into the adult stage and reduced adult lifespan (Table 4). In alignment with our findings, *S. pimpinellifolium*, even though possessing the highest number of nonglandular trichomes, has been reported to lack both antixenosis and antibiosis defences against *P. absoluta*.^17^, ^36^ Therefore, *S. arcanum* and *S. neorickii* could serve as valuable sources for breeding programmes aimed at developing resistant cultivars against *P. absoluta*.

Trichomes contribute to antibiosis and antixenosis types of resistance as they can delay pest acceptance, oviposition and larval settlement (nonglandular or glandular trichomes), or secrete secondary metabolites that impair pests during herbivory (glandular trichomes).^11^ We observed higher total abundances of trichomes in the wild species *S. arcanum* and *S. pimpinellifolium* and in the domesticated tomato Aurea F1 compared to other tomato genotypes. However, trichome density did not exert any effect on the oviposition of *P. absoluta* across all genotypes. Contradictory results on the effect of trichomes on *P. absoluta* oviposition are reported in the literature. A number of studies have reported no effect of glandular and nonglandular trichomes,^62^, ^63^ yet other studies reported a potential impact of trichomes, especially glandular trichomes on *P. absoluta* oviposition.^19^, ^31^, ^50^, ^56^ This disparity among studies might be a consequence of the fact that other factors such as volatile compounds play a significant role for host acceptance. For example, olfactory studies show that despite greater density of trichomes found on *S. habrochaites* and *S. pennellii*, *P. absoluta* was attracted to volatiles emitted by these tomatoes.^19^ Still, we observed longer larval settlement time on the wild tomato *S. arcanum*, which possessed by far the highest number of type I and IV glandular trichomes. This confirms the role of glandular trichomes in hindering *P. absoluta* larval settlement.^19^ Furthermore, the prolonged larval developmental time on Corona F1 and *S. neorickii*, the reduced larval survival on Corona F1, the lowered pupal weight on *S. arcanum* and *S. neorickii*, and reduced leaf damage on *S. arcanum* and *S. neorickii* could result from the presence of secondary metabolites produced by glandular trichomes. While *S. arcanum* exclusively possesses type I and IV glandular trichomes, Corona F1 and *S. neorickii* had higher levels of types VI and VII. Previous studies also have reported that these trichome types provide resistance against *P. absoluta*, primarily in wild species. For instance, acyl sugars found in the type IV glandular trichome in the wild accessions *S. galapagense* (VI037241) and *S. cheesmaniae* (VI037240) confer resistance to *P. absoluta*.^56^ Likewise, higher glandular trichome density, especially the presence of type I, IV and VI trichomes in the wild species *S. chilense*, *S. pimpinellifolium*, *S. cheesmaniae*, *S. galapagense*, *S. habrochaites* and *S. pennellii* was found to enhance resistance against *P. absoluta* by involving compound releases (e.g. zingiberene, acyl sugars, 2‐undecanone and 2‐tridecanone, and other allelochemicals).^31^, ^50^, ^64^ Therefore, type I, IV and VI trichomes in wild tomato species are highly effective in conveying pest resistance, and identifying the associated genes could be beneficial for breeding programmes.

Our laboratory assays were conducted using detached leaves and leaflets. This method is commonly used in the laboratory to avoid environmental influences experienced in glasshouses,^65^ yet may have affected the response of the plants to *P. absoluta* attacks. By using leaflets, we mainly assessed constitutive defences and potentially overlooked induced defences. Although resistance to *P. absoluta* involves a combination of constitutive and induced defence,^53^ it also has been shown that *P. absoluta* infestation downregulates several compounds in induced defence responses, including jasmonic acid, salicylic acid and total phenolic contents.^18^ Therefore, our findings still add to our understanding of the complex interactions between *P. absoluta* and its host plant.

Among the tested domesticated varieties, it is intriguing that Corona F1 shows effects on *P. absoluta* comparable to the wild species *S. arcanum* and *S. neorickii* despite the lower density of glandular trichomes. Our results thus contradict numerous assertions that domestication is systematically associated with the loss of traits involved in plant defence, leading to an increased susceptibility towards herbivorous insects.^19^, ^31^, ^48^ This is in accordance with Rowen and Kaplan,^3^ who reported that domesticated species can even provide stronger defence mechanisms during herbivory compared to their wild counterparts.

## 5 CONCLUSIONS

In summary, the evaluation of 19 tomato genotypes, including both domesticated varieties and wild species, revealed distinct patterns in trichome distribution and density and differences in their susceptibility to *P. absoluta* attacks. Our findings indicate that factors beyond trichome density, such as secondary metabolites, might contribute to pest resistance in both domesticated and wild tomatoes. This study not only enhances our understanding of plant–insect interactions, but also provides the initial steps for the development of more effective and sustainable pest control strategies. To complement those steps, further studies under semi‐field or field conditions using whole tomato plants instead of detached leaves are needed to cover the complex host‐searching and selection behaviour of adult *P. absoluta*. Moreover, an extended larval period in *P. absoluta* could increase the insect's susceptibility to attacks by natural enemies, secondary metabolites could influence survival and development of natural enemies, and trichome density could affect their searching behaviour and walking speed. Further investigations are therefore necessary to explore the interaction between tomato genotypes with varying levels of resistance and the natural enemies of *P. absoluta*, especially in the context of IPM.

## CONFLICT OF INTEREST

The authors declare that they have no conflicts of interests.

## Supporting information


**Table S1.** Trichome types and density (number of trichomes/24 mm^2^/leaflet) on the 19 tomato genotypes used in the experiments (sample size *n* = 8). All data are presented as mean ± SE.

## Data Availability

The data supporting this study's findings can be found at the figshare repository (see DOI: 10.6084/m9.figshare.26381440).
