# Supplementary material for: Response of the tomato leaf miner Phthorimaea absoluta to wild and domesticated tomato genotypes
Source: Pest Manag Sci. 2024 Nov 12;81(3):1345–59. doi: 10.1002/ps.8534 (PMC11821476; doi:10.1002/ps.8534)
Supplement: Supplementary file 1 — Table S1. Trichome types and density (number of trichomes/24 mm2/leaflet) on the 19 tomato genotypes used in the experiments (sample size n = 8). All data are presented as mean ± SE. [file PS-81-1345-s001.docx]

Perspective on the tomato leaf miner *Tuta absoluta* management: Insights from wild and domesticated tomato genotypes

Ayomide Joseph Zannou, Jörg Romeis, Jana Collatz

Agroscope, Research Division Agroecology and Environment, Reckenholzstrasse 191, 8046 Zurich, Switzerland

Corresponding author

Jana Collatz

Email: [jana.collatz@agroscope.admin.ch](mailto:jana.collatz@agroscope.admin.ch)

**Supplementary table 1.** Trichome types and density (number of trichomes/24mm^2^/leaflet) on the 19 tomato genotypes used in the experiments (sample size n = 8). All data are presented as mean ±SE.

|  | Upper side | | Lower side | | Glandular trichome types | | Non-glandular trichome types | |
| --- | --- | --- | --- | --- | --- | --- | --- | --- |
| Genotypes | Glandular | Non glandular | Glandular | Non glandular | I and IV | VI and VII | II and III | V and VIII |
| *S. lycopersicum* | | | | | | | | |
| Starbuck F1 | 15.4 ±3.99 hi | 56.0 ± 6.70 h | 11.4 ± 3.12 jk | 247.6 ± 9.77 n | 0.0 ± 0.0 b | 26.7 ± 6.88 g | 10.0 ± 1.85 ef | 293.6 ± 12.09 m |
| Tomamiro Muchoo | 17.1 ± 7.98 gh | 58.4 ± 7.00 gh | 14.9 ± 3.65 i | 249.5 ± 21.49 n | 0.0 ± 0.0 b | 32.0 ± 11.01 f | 7.2 ± 0.88 hi | 300.6 ± 27.90 l |
| Admiro F1 | 31.1 ± 6.39 d | 95.1 ± 22.80 c | 23.9 ± 2.22 de | 331.7 ± 43.34 g | 0.0 ± 0.0 b | 55.0 ± 7.96 c | 6.4 ± 0.88 ij | 420.5 ± 64.82 f |
| Cindel F1 | 4.5 ± 1.58 j | 34.7 ± 4.53 j | 4.6 ± 0.96 m | 223.1 ± 19.22 o | 0.0 ± 0.0 b | 9.1 ± 1.78 i | 12.5 ± 1.72 d | 245.4 ± 22.29 o |
| Berner Rose | 30.0 ± 4.84 de | 72.7 ± 7.79 f | 11.5 ± 2.63 jk | 316.1 ± 20.75 i | 0.5 ± 0.33 b | 41.0 ± 6.83 e | 8.1 ± 0.93 ghi | 380.7 ± 24.39 gh |
| Corona F1 | 37.9 ± 6.06 c | 62.0 ± 8.28 g | 26.0 ± 6.24 cd | 447.0 ± 85.63 b | 0.4 ± 0.26 b | 63.5 ± 9.92 b | 10.9 ± 1.59 e | 498.1 ± 88.55 c |
| Aurea F1 | 43.7 ± 6.11 b | 78.1 ± 6.11 e | 21.9 ± 3.72 ef | 470.9 ± 25.68 a | 0.1 ± 0.12 b | 65.5 ± 7.22 b | 8.1 ± 0.81 ghi | 540.9 ± 31.17 b |
| Romabelle F1 | 10.6 ± 2.42 i | 60.5 ± 10.43 gh | 7.4 ± 1.33 l | 308.6 ± 16.27 j | 0.6 ± 0.62 b | 17.4 ± 3.54 h | 9.5 ± 1.55 efg | 359.6 ± 24.09 i |
| Goldene Königin | 15.1 ± 6.84 hi | 86.1 ± 12.30 d | 8.6 ± 2.30 kl | 321.7 ± 17.51 h | 0.0 ± 0.0 b | 23.7 ± 8.27 g | 21.1 ± 1.41 a | 386.7 ± 26.37 g |
| Beorange F1 | 42.7 ± 10.08 b | 44.1 ± 6.60 i | 20.5 ± 4.20 fg | 278.9 ± 23.31 l | 0.0 ± 0.0 b | 63.2 ± 12.78 b | 8.0 ± 1.64 ghi | 315.0 ± 28.12 k |
| Indalo F1 | 27.0 ± 7.61 e | 97.4 ± 20.55 c | 19.2 ± 4.11 fgh | 355.5 ± 29.21 f | 0.1 ± 0.12 b | 46.1 ± 11.48 d | 5.6 ± 0.92 j | 447.2 ± 48.67 e |
| Green Zebra | 14.6 ± 4.98 hi | 37.7 ± 6.06 j | 17.7 ± 4.03 ghi | 255.0 ± 14.60 m | 0.0 ± 0.0 b | 32.4 ± 8.21 f | 7.7 ± 1.01 ghi | 285.0 ± 16.83 n |
| Costoluto Genovese F1 | 20.5 ± 4.53 fg | 42.2 ± 4.71 i | 12.4 ± 1.43 j | 171.5 ± 22.00 p | 0.0 ± 0.0 b | 32.9 ± 4.72 f | 7.1 ± 0.58 hi | 206.6 ± 25.47 p |
| Previa F1 | 22.8 ± 3.87 f | 82.7 ± 15.46 d | 16.6 ± 2.92 hi | 376.5 ± 39.69 e | 0.0 ± 0.0 b | 39.5 ± 5.83 e | 9.4 ± 1.42 efg | 449.9 ± 52.99 e |
| Noire de Crimée | 36.0 ± 11.36 c | 108.1 ± 18.17 b | 26.9 ± 6.77 c | 397.1 ± 40.23 d | 0.1 ± 0.12 b | 62.7 ± 16.24 b | 14.2 ± 1.65 c | 491.0 ± 55.59 d |
| Rentita | 46.6 ± 9.73 b | 70.6 ± 22.35 f | 27.6 ± 1.91 c | 286.2 ± 63.77 k | 0.4 ± 0.18 b | 73.9 ± 11.14 a | 8.6 ± 1.19 fgh | 348.2 ± 84.60 j |
| *S. pimpinellifolium* | 12.1 ± 3.81 i | 132.9 ± 21.04 a | 21.2 ± 3.73 ef | 430.2 ± 55.33 c | 1 ± 0.73 b | 32.4 ± 6.50 f | 9.9 ± 0.97 ef | 553.2 ± 75.88 a |
| *S. arcanum* | 284.5 ± 24.98 a | 0.0 ± 0.0 | 495.9 ± 27.24 a | 0.0 ± 0.0 q | 762.0 ± 44.37 a | 18.4 ± 3.24 h | 0.0 ± 0.0 k | 0.0 ± 0.0 q |
| *S. neorickii* | 12.5 ± 1.58 hi | 62.6 ± 3.80 g | 32.1 ± 5.52 b | 333.7 ± 22.46 g | 0.0 ± 0.0 b | 44.6 ± 6.47 d | 19.6 ± 2.33 b | 376.7 ± 23.51 h |
| χ2 | 75.56 | 71.252 | 78.929 | 81.435 | 77.37 | 71.409 | 77.685 | 81.412 |
| Df | 18 | 18 | 18 | 18 | 18 | 18 | 18 | 18 |
| *P* | *** | *** | *** | *** | *** | *** | *** | *** |

Note: Means followed by different letters within a column are significantly different (Kruskal-Wallis test, followed by Dunn Test P < 0.001).
